# Magnetic Resonance Elastography for the Detection and Classification of Prostate Cancer

**DOI:** 10.3390/cancers16203494

**Published:** 2024-10-15

**Authors:** Seung Ho Kim, Joo Yeon Kim, Moon Jung Hwang

**Affiliations:** 1 Department of Radiology, Inje University College of Medicine, Haeundae Paik Hospital, Busan 48108, Republic of Korea; 2 Department of Pathology, Inje University College of Medicine, Haeundae Paik Hospital, Busan 48108, Republic of Korea; h00311@paik.ac.kr; 3 Advanced Medical Imaging Institute, Korea University Anam Hospital, Seoul 02841, Republic of Korea; mrphd@korea.ac.kr

**Keywords:** magnetic resonance imaging (MRI), magnetic resonance elastography (MRE), prostate gland, prostate cancer

## Abstract

We investigated the feasibility of magnetic resonance elastography (MRE) using a pelvic acoustic driver for the detection and classification of prostate cancer (PCa). A total of 75 consecutive patients (mean age, 70; range, 56–86) suspected of having PCa and who underwent multi-parametric MRI including MRE and subsequent surgical resection were included. The analyzed regions consisted of cancer (n = 69), benign prostatic hyperplasia (BPH) (n = 70), and normal parenchyma (n = 70). A histopathologic topographic map served as the reference standard for each region. One radiologist and one pathologist performed radiologic–pathologic correlation, and the radiologist measured stiffness values in each region of interest on elastograms automatically generated by dedicated software. Paired t-tests were used to compare stiffness values between two regions. ROC curve analysis was also used to extract a cutoff value between two regions. The stiffness value of PCa (unit, kilopascal (kPa); 4.9 ± 1.1) was significantly different to that of normal parenchyma (3.6 ± 0.3, *p* < 0.0001) and BPH (4.5 ± 1.4, *p* = 0.0454). Under a cutoff value of 4.2 kPa, a maximum accuracy of 87% was estimated, with a sensitivity of 73%, a specificity of 99%, and an AUC of 0.839 for discriminating PCa from normal parenchyma. Between PCa and BPH, a maximum accuracy of 62%, a sensitivity of 70%, a specificity of 56%, and an AUC of 0.598 were estimated at a 4.5 kPa cutoff. The stiffness values tended to increase as the ISUP grade increased. In conclusion, it is feasible to detect and classify PCa using pelvic MRE. Our observations suggest that MRE could be a supplement to multi-parametric MRI for PCa detection.

## Simple Summary

Multi-parametric MRI is a first-line imaging modality for prostate cancer detection. Magnetic resonance elastography (MRE) is a useful tool for measuring parenchymal stiffness to diagnose liver fibrosis. A known physical characteristic of prostate cancer is that it is harder than a normal prostate gland. We hypothesized that the quantitative stiffness value derived from MRE could be used to discriminate prostate cancer from benign prostate hyperplasia and normal parenchyma. Until now, the application of MRE to prostate cancer has been sporadic; it has only been used for investigational purposes due to the uncomfortable perineal placement of the acoustic device and inconvenience to patients during preparation. Therefore, we attempted to place an acoustic driver on the lower abdominal wall and determined that detecting and classifying prostate cancer via MRE is feasible. Our observation of 75 consecutive patients revealed that the stiffness value of prostate cancer was significantly different from normal parenchyma, and an accuracy of 87% was estimated at a cutoff value of 4.2 kPa in discriminating prostate cancer from normal parenchyma. In terms of differentiating prostate cancer from benign prostate hyperplasia, an accuracy of 62% was estimated. Additionally, the stiffness values tended to increase as the ISUP grade increased. This observation suggests that the stiffness value derived from pelvic MRE is helpful for detecting and classifying prostate cancer and could be an adjunct to multi-parametric MRI.

## 1. Introduction

Multi-parametric magnetic resonance imaging (MRI), including T2-weighted imaging (T2WI) and diffusion-weighted imaging (DWI), is a first-line imaging modality in the detection and local staging of prostate cancer (PCa) [1,2,3]. Management of PCa is individually tailored by several factors such as the patient’s age, prostate specific antigen (PSA) level, and Gleason score (GS). In particular, GS is a PCa classification system based on the structure of PCa and is related to tumor aggressiveness. Combining of Gleason scores into a three-tiered grouping (6, 7, 8–10) is used most frequently for prognostic and therapeutic purposes [4].

Recent advances in MRI include techniques such as diffusion, perfusion, and texture for acquisition of the physical properties of specific tissues [5,6,7]. Magnetic resonance elastography (MRE) is an emerging technique for measuring liver stiffness and has shown promising results for staging liver fibrosis [8,9,10]. Moreover, the prostate gland is one of the organs other than the liver in which MRE could be implemented, considering the previous observation that PCa causes the prostate to have a higher stiffness value than the normal prostate gland [11,12,13].

MRE is a non-invasive method that can evaluate the mechanical properties of tissues, making it useful for diagnosing and monitoring various diseases. The basic principle of MRE is to transmit external mechanical vibrations to the tissue and measure the resulting displacements to calculate tissue stiffness. As tissue stiffness varies, the phase values of the magnetic resonance (MR) signal change accordingly—stiffer tissues result in greater phase shifts. These phase changes are then inverted to calculate stiffness values. Accurate calculation of these values relies heavily on high signal-to-noise ratios in the base images and phase-contrast imaging. Effective transmission of vibrations and visualization of these small changes are crucial for evaluating the mechanical properties of tissues non-invasively.

Previous studies on prostate gland MRE have used a passive acoustic driver placed in the patient’s urethra, rectum, or perineum [14,15,16]. However, the uncomfortable placement of the acoustic device and inconvenience to patients during preparation is an issue. Therefore, we attempted to place an acoustic driver on the lower abdominal wall and investigated whether this could detect and classify PCa. To our knowledge, this has rarely been attempted for PCa [17,18,19]; moreover, studies have used relatively small populations and systematic biopsy as the gold standard rather than pathologic whole mount after radical prostatectomy. Our hypothesis was that the quantitative stiffness value derived from MRE could be used to discriminate PCa from benign prostatic hyperplasia (BPH) and normal parenchyma. Thus, the aim of this study was to investigate the feasibility of MRE combined with a pelvic acoustic driver for the detection and classification of PCa with topographic maps as the reference standard.

## 2. Materials and Methods

Our institutional review board approved this study (IRB 2024-03-016-002), and informed consent was waived due to the extremely low risk to patients associated with this retrospective study. 

### 2.1. Patient Selection Criteria

**Figure 1:**
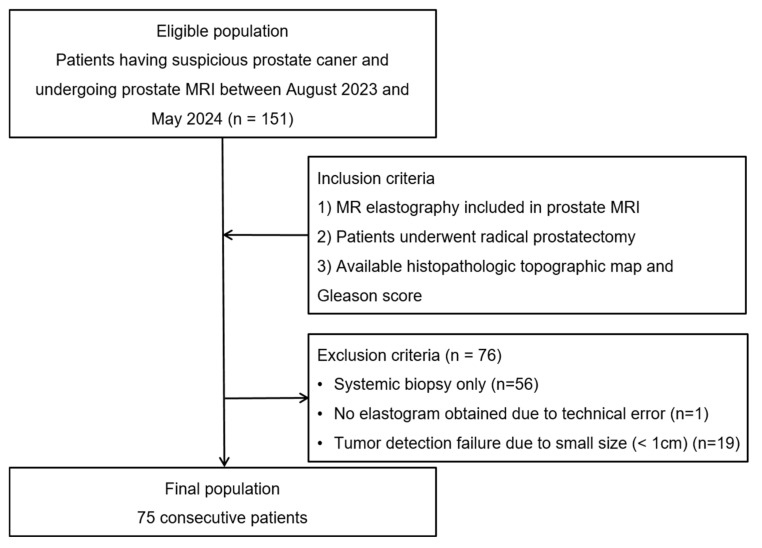
Flowchart of the case-accrual process.

A total of 151 patients who had suspected PCa and underwent prostate MRI between August 2023 and May 2024 were initially eligible for this study. Patients who satisfied the following inclusion criteria were selected: (1) patients who underwent MRE; (2) patients who underwent subsequent robot-assisted laparoscopic radical prostatectomy; (3) patients who had histopathologic whole mount and relevant full pathologic information including GS. From this population, 56 patients who had undergone systematic biopsy only were excluded. In addition, 19 patients whose tumor size was less than 1 cm were excluded due to the inherent limitations of MRE in detecting small tumors. One patient was excluded due to technical errors during MRE. Finally, 75 patients (mean age, 70; range, 56–86) were included and analyzed. The patient accrual process is presented in Figure 1. 

### 2.2. Magnetic Resonance Imaging

All MRI scans were performed using a 3.0-T MR machine (Signa Architect, GE HealthCare, Chicago, IL, USA) with a torso coil (AIRTM Anterior Array, GE HealthCare Coils, Aurora, OH, USA). The scan protocol comprised bi-parametric MRI including T2WI in the axial, sagittal, and coronal planes, axial DWI sequences (b-values of 0, 100, 1000, and 2000 s/mm^2^), and corresponding apparent diffusion coefficient (ADC) maps.

### 2.3. Magnetic Resonance Elastography

The MRE system consisted of active and passive drivers. The active driver (RESOUNDANT, benchmark electronics Inc. Rochester MN, USA) generates continuous acoustic vibrations at a 60 to 120 Hz frequency, transmitted via a flexible vinyl tube to the passive driver positioned on the patient’s lower abdomen. The passive driver converts these vibrations into mechanical waves that propagate through the tissues. These waves are synchronized with a modified two-dimensional (D) spin-echo (SE) echo planar imaging (EPI) pulse sequence, allowing phase-contrast imaging to measure tissue displacements. Since these displacements are proportional to tissue stiffness, this method provides valuable relative stiffness information. 

A 2D SE EPI-based phase-contrast sequence was employed to capture the phase displacements correlated with tissue stiffness. The pulse sequence was modified with an oscillating motion-encoding gradient (MEG), applied in the slice direction at 90 Hz. The MEG duration matched the mechanical vibration period, leading to phase shifts in the MR signal corresponding to mechanical excursions. These phase shifts were inverted voxel by voxel to calculate stiffness values using the multi-model direct inversion (MMDI) algorithm. Given that the prostate is a small structure, and to differentiate the lesions within it, relatively shorter wavelengths are required. However, using higher frequencies to achieve shorter wavelengths results in reduced penetration depth, making it challenging for the vibrations to effectively reach the prostate. Therefore, under the current imaging conditions and available MR hardware, a 90 Hz frequency was determined to be optimal for clinical application to the prostate. Future improvements in MR hardware or pulse sequences could allow for effective imaging with even shorter wavelengths.

The MRE acquisition parameters were a TR/TE of 2000/70.5 ms, FOV of 240 mm, matrix size of 80 × 80, and slice thickness of 3.0 mm, with a reconstructed voxel size of 1.5 × 1.5 × 3.0 mm. The imaging sequence utilized chemical fat saturation, a number of excitations (NEX) of 8, a bandwidth of 250 kHz, a temporal phase of 4, an MEG and driver frequency of 90 Hz in the slice direction, and a free-breathing scan time of 2 min and 16 s. The scan parameters are presented in detail in Table 1. 

### 2.4. Image Analysis

Data processing was performed using MR-touch in ReadyView, which is installed within the AW system (AW server 3.2 Ext. 4.9, GE HealthCare). Region-of-interest (ROI) measurements were taken from color-coded elastograms (maximum 8 kilopascals) fused with T2WI using rigid registration, as the elastogram images alone were not sufficient for viewing small anatomical structures. Using the fused images of T2WI and stiffness maps, along with wave images for reference, a freehand ROI was employed to measure the whole tumor volume by tracing the tumor margin across all visible slices.

### 2.5. Reference Standard

All patients underwent robot-assisted radical prostatectomy. Resected specimens were evaluated by a dedicated pathologist. Histopathologic topographic maps served as the reference standard for the radiologic–pathologic correlation of PCa. 

### 2.6. Statistical Analysis

All the statistical analyses were performed with MedCalc software for Windows (MedCalc Software version 22.032, Mariakerke, Belgium). When the calculated *p*-value was less than 0.05, it was considered a statistically significant difference. Paired t-tests were used to compare stiffness values between two regions. Receiver operating characteristic (ROC) curve analysis was also used to extract an optimal cutoff value between two regions. The area under the ROC curve (AUC) was calculated and represented diagnostic performances.

## 3. Results

### 3.1. Patient Demographics

The analyzed regions consisted of cancer (n = 69), benign prostatic hyperplasia (BPH) (n = 70), and normal parenchyma (n = 70). Six patients had high grade prostatic intraepithelial neoplasia, which is considered as the precursor of PCa. The mean interval from preoperative prostate MRI and radical prostatectomy was 58 days (range: 7–137 days), and the mean PSA level was 21.0 ng/mL (range: 0.25–527). Patients were grouped according to International Society of Urologic Pathologists (ISUP) grades and GSs as follows: ISUP grade 1 (GS6, n = 18), ISUP grade 2 (GS3 + 4, n = 24), ISUP grade 3 (GS4 + 3, n = 19), ISUP grade 4 (GS8, n = 4), and ISUP grade 5 (GS9, n = 4). PCa was not found in six patients. The tumor locations included the peripheral zone (PZ, n = 35), transition zone (TZ, n = 22), diffuse involvement (n = 10), and anterior fibrous stroma (n = 2). The detailed demographic data are summarized in Table 2. 

### 3.2. Comparison of Stiffness Values among Three Groups

**Figure 2:**
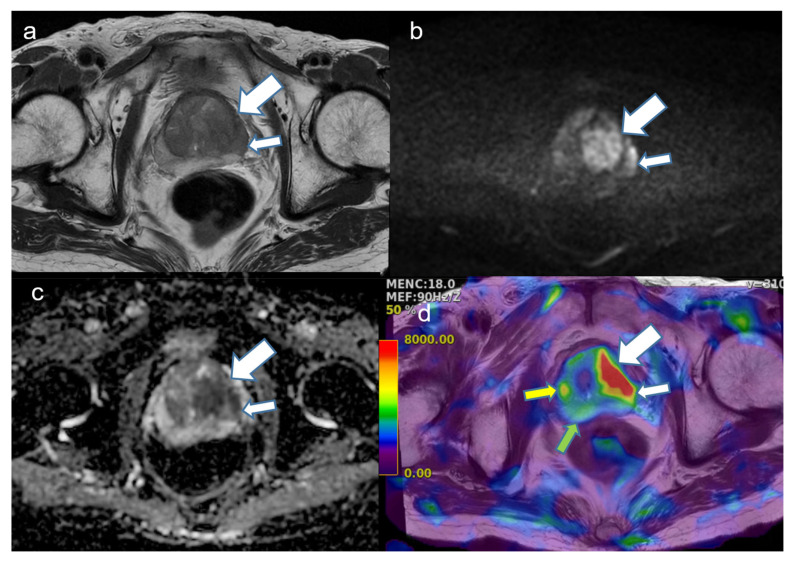
A representative case of a 72-year-old man with prostate cancer with a Gleason score of 7 (4 + 3) in the transition zone. (**a**) T2-weighted image shows a 3 cm low-signal-intensity (SI) lesion (large arrow) in the left transition zone (TZ) with an extension (small arrow) to the neighboring peripheral zone (PZ). It is difficult to demarcate low SI in the benign prostatic hyperplasia (BPH) nodule in the right TZ. (**b**) Diffusion-weighted image (b value, 2000 s/mm^2^) showing a heterogeneous high SI. (**c**) Corresponding apparent diffusion coefficient map also showing a reciprocal low SI in the tumor. Some diffusion-restricted areas are also seen in the BPH nodule in the right TZ. (**d**) Elastogram fused with T2-weighted image showing a high stiffness value (red area, white arrow) in the tumor. Compared with the stiffness value of 7.3 kilopascals in the tumor, the BPH nodule (yellow arrow) was measured at 4.1 kilopascals and normal parenchyma in the PZ (green arrow) indicated 3.2 kilopascals.

The stiffness value for PCa (unit, kilopascal; 4.9 ± 1.1) was significantly higher than that for normal parenchyma (3.6 ± 0.3, *p* < 0.0001) and BPH (4.5 ± 1.4, *p* = 0.0454) (Figure 2). At a 4.2 kPa cutoff, a maximum accuracy of 87% was estimated, with a sensitivity of 73%, a specificity of 99%, and an AUC of 0.839 for discriminating PCa from normal parenchyma. Between PCa and BPH, a maximum accuracy of 62%, a sensitivity of 70%, a specificity of 56%, and an AUC of 0.598 were estimated at a 4.5 kPa cutoff.

### 3.3. Comparison of Stiffness Values According to Tumor Location

**Figure 3:**
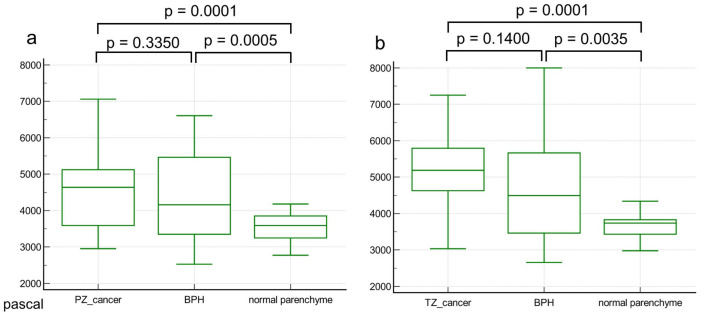
Box and whisker plots comparing the stiffness values of prostate cancer, benign prostatic hyperplasia (BPH), and normal parenchyma according to tumor location. For both peripheral-zone (**a**) and transition-zone cancers (**b**), the stiffness value of prostate cancer was higher than normal parenchyma; however, it did not show a significant difference compared to BPH. The middle line in each box represents the median. The lower and upper boundaries of the boxes represent the lower and upper quartiles (25th and 75th percentiles, respectively). The whiskers indicate the range from the maximum to the minimum calculated stiffness values in pascals.

In PZ cancer, the stiffness value for PCa (4.6 ± 1.1) was higher than that for normal parenchyma (3.6 ± 0.3, *p* = 0.0001) but was not significantly different to that for BPH (4.4 ± 1.2, *p* = 0.3350). A similar trend was observed for TZ cancer between PCa (5.2 ± 1.0) and normal parenchyma (3.7 ± 0.3, *p* < 0.0001) as well as BPH (4.5 ± 1.6, *p* = 0.1400). Box and whisker plots are presented in Figure 3, and the detailed results are summarized in Table 3.

### 3.4. Comparison of Stiffness Values According to ISUP Grade Group

**Figure 4:**
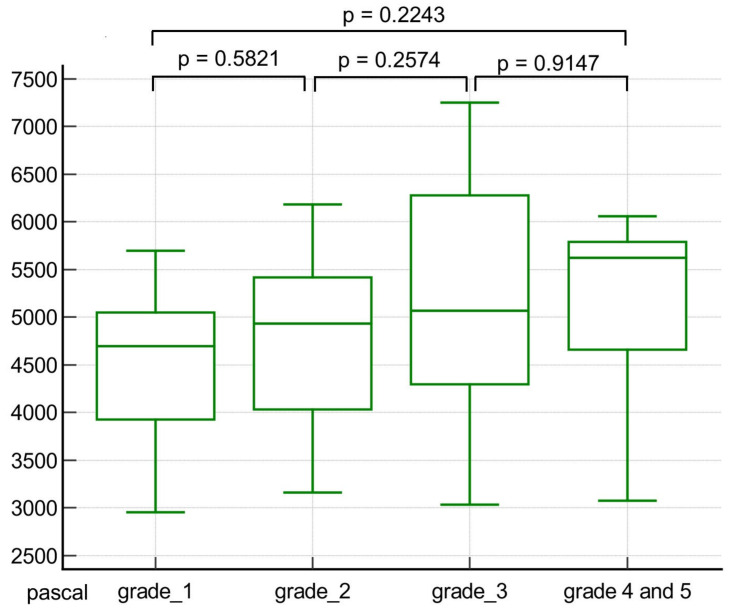
A box and whisker plot comparing the stiffness values of prostate cancer according to International Society of Urologic Pathologists (ISUP) grade group. The mean stiffness value of prostate cancer shows an increasing tendency as ISUP grade increases. The middle line in each box represents the median. The lower and upper boundaries of the boxes represent the lower and upper quartiles (25th and 75th percentiles, respectively). The whiskers indicate the range from the maximum to the minimum calculated stiffness values in pascals.

Stiffness values tended to increase as the ISUP grade increased. The mean stiffness values between grade 1 (4.5 ± 0.8) and grades 4 and 5 (5.1 ± 1.0) were not significantly different (*p* = 0.2243). This was also true between grade 1 and grade 2 (4.7 ± 1.0, *p* = 0.5821) and between grade 2 and grade 3 (5.2 ± 1.4, *p* = 0.2574). A box and whisker plot is presented in Figure 4, and the detailed results are described in Table 4. 

## 4. Discussion

Our study found that the stiffness value for PCa was higher than that for normal parenchyma, regardless of location. Our observation corresponds well with previous studies [13,18,19]. Previous studies have reported that the stiffness of PCa was higher than that of normal parenchyma. Li et al. reported that the shear wave velocity that corresponds to the tissue stiffness value for PCa was higher than that for normal parenchyma (unit, m/sec, 3.4 ± 06, 2.2 ± 0.1, *p* < 0.001) according to multi-frequency MRE [19]. In addition, this finding has also been observed with sonoelastography [11,12]. The stiffness of PCa was also found to be higher than that of normal parenchyma (unit, kPa, 8.7 ± 3.4, 3.6 ± 1.3) in a small study population (less than 10 patients in each group).

The difference between PCa and normal parenchyma can be explained from a histologic perspective [20,21,22,23]. The epithelium–stroma interaction has a major role in maintaining the structure and function of normal tissue. In carcinomas, this balance is disrupted, leading to the generation of an abnormal tumor microenvironment consisting of fibroblasts, immune cells, and extracellular matrix proteins [20]. Cancer cells interact with these stromal components through many signaling factors [21]. In particular, cancer-associated fibroblasts (CAFs) play an essential role in PCa by overproducing various extracellular matrix components such as collagens, consequently increasing matrix stiffening and thereby promoting tumor growth and invasion [22,23,24].

Although the overall results comparing PCa and BPH showed a difference in the stiffness values, our subgroup analyses found that the stiffness values for PCa were not different from those for BPH in both PZ and TZ. These results differ from those of previous studies [18,19,25], which observed that the stiffness of PCa was higher than that of BPH. Li et al. observed that TZ cancers had a higher shear wave velocity (stiffness value) than did BPH (unit, m/s; 3.1 ± 0.4, 2.6 ± 0.3, *p* < 0.001) [19]. Lee et al. also reported stiffness values for PCa and BPH nodules (unit, kPa; 5.99 ± 1.46, 4.67 ± 1.54, *p* = 0.045) based on systematic biopsy rather than radical prostatectomy specimens [25]. However, these studies have limited generalizability due either to their small study population (19 PCa and 10 BPH nodules) [25] or their gold standard being based on cognitive fusion biopsy rather than histologic topographic maps of the excised specimens [19].

**Figure 5:**
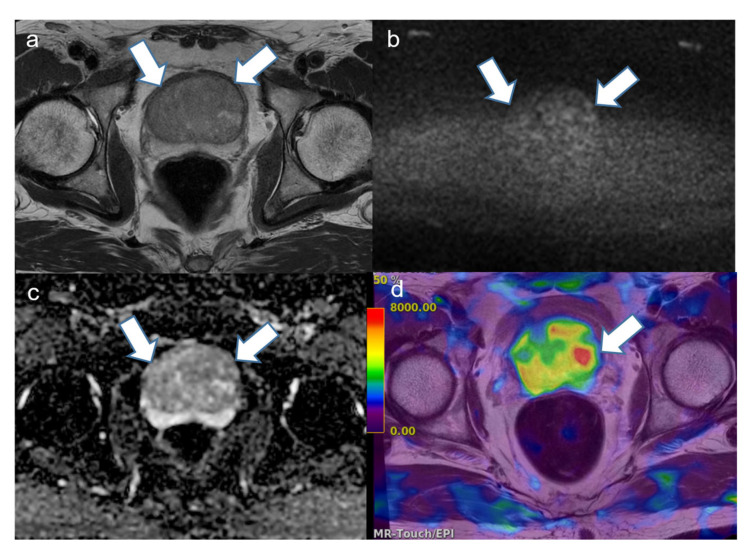
A representative case of a 73-year-old man with a benign prostatic hyperplasia (BPH) nodule (arrows) showing the internal heterogeneity of the stiffness value. (**a**) T2-weighted image shows a large BPH nodule with heterogeneous signal intensity (SI) in the transition zone. (**b**) Diffusion-weighted image (b value, 2000 s/mm^2^) showing a heterogeneous high SI in the BPH nodule. (**c**) The corresponding apparent diffusion coefficient map also shows a reciprocal heterogeneous low SI in the BPH nodule. (**d**) Elastogram fused with T2-weighted image shows a wide range of stiffness values in the BPH nodule. Some portions indicate a stiffness value of 8 kilopascals, which is similar to that of prostate cancer.

Therefore, the substantial overlap of stiffness values between PCa and BPH might have been overlooked in previous studies. In our opinion, the discrepancy with previous studies could be explained by two factors: the measurement method for ROIs and the heterogeneity of PCa and BPH. In terms of measuring the stiffness values of focal prostate lesions including PCa and BPH, the previous studies have used either one single slice covering the focal lesion [25] or three consecutive slices [19]. However, this method could not represent the whole BPH nodule volume. To overcome this drawback, we used the whole volume measurement method instead, which is known to be robust and the most reproducible among the various quantitative measurement methods in the field of oncology [26]. Based on our observation, we suggest that the wide range of stiffness values for BPH could be attributed to some overlap with PCa. Our observation is supported by a recent study [27] by Reiter et al., who also reported that PCa is characterized by a homogeneous stiff biomechanical signature, possibly due to the unique nondestructive growth pattern of PCa with intervening stroma. Increased heterogeneity in neighboring BPH was also observed [27]. The larger the BPH nodule, the wider the range of heterogeneity of the BPH nodule. In this regard, we suggest that the stiffness value measured by MRE has an inherent limitation in discriminating PCa from BPH, particularly in the TZ (Figure 5). Our opinion can be supported by the histopathologic explanation of BPH. Histological BPH can be defined as epithelial and stromal proliferation in the prostate TZ, with a predominance of stromal cells. Compared with normal prostatic tissue, the balance between the growth and apoptosis of stromal cells in hyperplastic nodules is lost, resulting in an increase in stromal volume, which leads to the increased stiffness of BPH [28].

In terms of differentiating ISUP grade groups, the diagnostic performance of the imaging technique is important in treatment planning and risk stratification in patients with PCa. Various MRI techniques have been used to discriminate clinically significant cancer (CSC, GS ≥ 7) from non-CSC (GS6) [5,6,7]. Our study revealed that the stiffness value tended to increase as the ISUP grade increased. Similar results were reported in a previous study [13]. Reiter et al. also observed a similar increasing tendency for stiffness as the GS increased from GS6 to GS8/9. However, the diagnostic task of discriminating CSC from non-CSC is challenging when using other imaging modalities, in light of the inherent limitations in analyzing the tumor microenvironment. This is the case with MRE. Based on our observation, the potential use of MRE to discriminate ISUP grade 2 (GS 3 + 4) from grade 3 (GS 4 + 3) is also limited.

In this study, we validated the technical feasibility of MRE with a pelvic acoustic driver. In contrast to the other acoustic drivers used in previous studies that may cause discomfort associated with their endo-rectal, perineal, or transurethral placement and inconvenience to patients during preparation [14,15,16], the placement of the acoustic driver on the low abdominal wall can be easily adopted and incorporated into clinical practice, with good compliance of patients.

Several limitations in our study should be acknowledged. First, there could be selection bias associated with the retrospective study design. Patients undergoing systemic biopsy were excluded in this study. However, radical prostatectomy was necessary to obtain a topographic map, which was a ground truth for the segmentation of PCa, BPH, and normal parenchyma. We believe that the histologic whole mount is an ideal guide for radiologic–pathologic correlation. Moreover, measurement was performed based on a relatively large study population compared with previous studies, which is a merit of this study. Another limitation is that we excluded small PCa (less than 1 cm) from further analyses based on our experience and previous observations [25]. In this study, we used a high frequency of 90 Hz for prostate imaging, which provided shorter wavelengths and better resolution, which is crucial for assessing smaller structures. However, it was challenging to measure tumors smaller than 1 cm with MRE. This limitation suggests that using frequencies higher than 90 Hz to produce shorter wavelengths could improve resolution and potentially solve this issue. However, higher frequencies result in reduced penetration depth, making it difficult for the vibrations to effectively reach the prostate gland. Lastly, we did not compare the diagnostic performance of MRE with that of other techniques, including DWI and corresponding ADC maps, for specific tasks such as discriminating CSC from non-CSC. It would be intriguing to compare the diagnostic efficacy of MRE with that of DWI and corresponding ADC for incorporation into clinical practice. However, this task is beyond the scope of this study; thus, we focused on the feasibility of MRE with a pelvic acoustic driver. Further efficacy studies are warranted. 

## 5. Conclusions

In conclusion, it is feasible to detect PCa via MRE with a pelvic acoustic driver. Our observations suggest that MRE could be a supplement to multi-parametric MRI for PCa detection. In addition, the wide range of stiffness values for BPH could be attributed to the substantial overlap between PCa and BPH.

## Figures and Tables

**Table 1:** MRI sequence parameters.

Parameter	T2WI (Axial, Sagittal, and Coronal)	DWI (b = 0, 100, 1000 and 2000 s/mm^2^)	MR Elastography
TR	4680~4930	5260	2000
TE	75~100	88.5	70.5
ETL	15	2	2
Slice thickness	3.0 mm	3.0 mm	3.0 mm
Slice gap	0.3 mm	0.3 mm	0.3 mm
Matrix size (axial)	400 × 320	120 × 120	80 × 80
NEX	1	2, 2, 4, 8	8
FOV (mm)	220 × 220	240 × 240	240 × 240
Acquisition time	1 min 28 s~1 min 51 s	7 min 53 s	2 min 16 s

Repetition time (TR); Echo time (TE); Echo train length (ETL); Field of view (FOV); Number of excitations (NEX); Diffusion-weighted imaging (DWI); MR elastography was performed by utilizing 2-dimensional spin-echo echo planar imaging pulse sequence.

**Table 2:** Demographic data of the study population.

Parameter	Study Population (n = 75)
Mean age, years [range]	70 (56–86)
Mean PSA, ng/mL [range]	21.0 (0.25–527)
Mean interval from MRI to radical prostatectomy, days [range]	58 (7–137)
Gleason score of prostate cancer	
6	18
7	43
3 + 4	24
4 + 3	19
8	4
9	4
High grade prostatic intraepithelial neoplasia	6
Tumor location	
Peripheral zone	35
Transition zone	22
Anterior fibromuscular stroma	2
Diffuse	10

Prostate-specific antigen (PSA).

**Table 3:** Comparison of stiffness values of three groups according to cancer location.

Location	PZ Cancer		TZ Cancer	
	Stiffness Value	*p* Value	Stiffness Value	*p* Value
Prostate cancer	4.6 ± 1.1	0.3350 *	5.2 ± 1.0	0.1400 *
BPH	4.4 ± 1.2	0.0005 ^†^	4.5 ± 1.6	0.0035 ^†^
Normal parenchyma	3.6 ± 0.3	0.0001 ^‡^	0.7 ± 0.3	0.0001 ^‡^

* comparison between prostate cancer and BPH; ^†^ comparison between BPH and normal parenchyma; ^‡^ comparison between prostate cancer and normal parenchyma; PZ cancer, peripheral zone cancer; TZ cancer, transition zone cancer; BPH, benign prostatic hyperplasia.

**Table 4:** Comparison of stiffness values according to ISUP grade group.

ISUP Grade	Stiffness Value (unit, kilopascal)	*p* Value
Grade 1 (GS 6)	4.5 ± 0.8	0.5821 *
Grade 2 (GS 3 + 4)	4.7 ± 1.0	0.2574 ^†^
Grade 3 (GS 4 + 3)	5.2 ± 1.4	0.9147 ^‡^
Gr.4 (GS8) & Gr.5(GS9)	5.1 ± 1.0	0.2243 ^§^

* comparison between Gr. 1 and Gr. 2; ^†^ comparison between Gr. 2 and Gr. 3; ^‡^ comparison between Gr. 3 and Gr. 4 and 5; ^§^ comparison between Gr. 1 and Gr. 4 and 5; Data are presented as mean ± standard deviations; ISUP, international society of urologic pathologists; GS, Gleason score.

## Data Availability

The data presented in this study are available on request from the corresponding author. The data are not publicly available due to patients’ privacy.
